# Median Urinary Iodine Concentrations Are Indicative of Adequate Iodine Status among Women of Reproductive Age in Prey Veng, Cambodia

**DOI:** 10.3390/nu8030139

**Published:** 2016-03-03

**Authors:** Crystal D. Karakochuk, Kristina D. Michaux, Tze L. Chai, Benny B. Chan, Kyly C. Whitfield, Susan I. Barr, Judy McLean, Aminuzzaman Talukder, Kroeun Hou, Sokhoing Ly, Tim J. Green

**Affiliations:** 1 Food, Nutrition and Health, The University of British Columbia, 2205 East Mall, Vancouver, BC V6T 1Z4, Canada; crystal.karakochuk@alumni.ubc.ca (C.D.K.); kristina.michaux@ubc.ca (K.D.M.); tlinchai@alumni.ubc.ca (T.L.C.); bennycha@mail.ubc.ca (B.B.C.); kyly@mail.ubc.ca (K.C.W.); susan.barr@ubc.ca (S.I.B.); judy.mclean@ubc.ca (J.M.); 2 Child and Family Research Institute, 950 West 28th Ave, Vancouver, BC V5Z 4H4, Canada; 3 Helen Keller International, P.O. Box 168, Phnom Penh 12301, Cambodia; ztalukder@hki.org (A.T.); hkroeun@hki.org (K.H.); lsokhoing@hki.org (S.L.); 4 South Australian Health and Medical Research Institute, and the Women’s and Children’s Health Research Institute, North Terrace, Adelaide 5000, Australia

**Keywords:** Cambodia, deficiency, iodine, urine, women

## Abstract

Iodine deficiency disorders are estimated to affect over 1.9 million people worldwide. Iodine deficiency is especially serious for women during pregnancy and lactation because of the negative consequences for both mother and infant. The aim of this cross-sectional study was to determine the median urinary iodine concentration (UIC) as a population-level indicator of iodine status among rural women farmers of reproductive age (18–45 years) in the province of Prey Veng, Cambodia. A total of 450 women provided a spot morning urine sample in 2012. Of those women, 93% (*n* = 420) were non-pregnant and 7% (*n* = 30) were pregnant at the time of collection. UIC was quantified using the Sandell-Kolthoff reaction with modifications. The median UIC of non-pregnant (139 μg/L) and pregnant women (157 μg/L) were indicative of adequate iodine status using the WHO/UNICEF/ICCIDD epidemiological criteria for both groups (median UIC between 100–199 and 150–249 μg/L, respectively). We conclude that non-pregnant and pregnant women in rural Prey Veng, Cambodia had adequate iodine status based on single spot morning urine samples collected in 2012. More research is warranted to investigate iodine status among larger and more representative populations of women in Cambodia, especially in light of recent policy changes to the national program for universal salt iodization.

## 1. Introduction

Iodine is an essential nutrient found in the human body as a key component of the thyroid hormones, thyroxine (T4) and triiodothyronine (T3). These thyroid hormones regulate cellular oxidation, energy metabolism, and the basal metabolic rate, and are critically important for neurological growth and development, particularly during gestation and early infancy [1,2]. Low iodine intake is associated with numerous negative health outcomes collectively called iodine deficiency disorders (IDD), which are estimated to affect over 1.9 million people worldwide [3]. The most common IDD in adults and children are hypothyroidism and goiter (enlarged thyroid gland), due to excessive secretion of thyroid stimulating hormone in response to low levels of circulating T4 resulting in an overactive thyroid gland [1,4]. Iodine deficiency in adults and adolescents is also associated with reduced work capacity and cognitive impairment [5]. Pregnant women and infants are particularly vulnerable to iodine deficiency given their increased iodine requirements. Severe iodine deficiency during gestation has the most detrimental effects, resulting in a condition called cretinism, characterized by irreversible physical and mental retardation in the newborn [2,4,5,6]. In addition to the consequences of iodine deficiency, excessive iodine intake is also a concern as it has been associated with negative outcomes such as impaired thyroid function [7,8]. As such, it is important to monitor the risk of both iodine deficiency and excess. The FAO/WHO have established daily recommended nutrient intakes (RNI) for each nutrient specified on the basis of age, gender and life stage to meet the nutrient requirements of ≥97.5% of a healthy population [7]. The RNI for iodine for healthy non-pregnant, non-lactating women ≥13 years through adulthood is 150 μg/day; however, during pregnancy and lactation, the RNI for women is increased to 250 μg/day [7,9]. The WHO also report that daily iodine intakes up to ~1000 μg/day appear to be safe [10].

Most commonly, median urinary iodine concentration (UIC) is used to assess population-level iodine status [1]. At the individual level, fluctuations in dietary iodine intake and fluid can result in high diurnal and within-day variation of UIC, which limit its use as a biomarker of individual iodine status [11]. Accordingly the WHO/UNICEF/ICCIDD recommend comparing the median UIC of a population to established reference criteria to assess iodine status as follows: Among non-pregnant women, a population median UIC <100 μg/L is indicative of insufficient iodine intake (iodine deficiency), 100–199 μg/L of adequate intake, 200–299 μg/L of intakes above requirements, and ≥300 μg/L of excessive intake. Among groups of pregnant women, a median UIC <150 μg/L is indicative of insufficient iodine intake, 150–249 μg/L of adequate intake, 250–499 μg/L of intakes above requirements, and ≥500 μg/L of excessive intake [11]. With regard to these reference ranges, it should be noted that the descriptor “intakes above requirements” is intended to convey that intakes are more than adequate, rather than to suggest that population medians below this range (in the “adequate intake” range) do not meet requirements.

The WHO/UNICEF/ICCIDD also recommends single spot urine samples for the assessment of population-level iodine status [11]. As compared to 24-h samples and/or the collection of multiple samples, spot samples are understandably easier to collect. A recent review on the iodine biomarkers by Rohner *et al.* [1] also concludes that UIC obtained from spot urine samples reflect 24-h intake in populations absent of acute malnutrition and that they are a reliable biomarker to assess population-level iodine status. However, we acknowledge that the recommendation for use of one or the other remains debatable by some in the field.

Iodine deficiency is endemic in some areas in Southeast Asia and most of West and Central Africa, especially those areas where soil levels are chronically depleted as a result of flooding and erosion [11,12]. In most of these areas, universal fortification of table salt was introduced as an inexpensive and safe method for the prevention and control of IDD, but despite these efforts, mild to moderate deficiency has remained or reemerged in countries previously considered iodine sufficient, such as Cambodia [13,14], potentially as a result of incomplete fortification and/or issues related to poor coverage or access of adequately iodized salt. A review paper on the iodine status of school-aged children using data collected between 1993 and 2012 showed the median UIC in Cambodia was between 200–299 μg/L, indicating more than adequate iodine status [3]. Another study by Perignon *et al.* [15] showed that a large proportion of children (~50%) from one province in Cambodia had an individual UIC of ≥200 μg/L, which is also suggestive of adequate to more than adequate status. We note these studies presented data for children only. Limited data on the iodine status of Cambodian women have been published prior to the most recent Cambodian Demographic and Health Survey (DHS) using data collected in 2013–2014. This survey suggested the possibility of mild to moderate deficiency among both women and children [16]. Although median UIC was not reported, 78% of women and 66% of children under 5 years of age had an individual UIC of <100 μg/L. Thus, median UIC would likely be <100 μg/L, consistent with mild deficiency among these groups at the population level.

The aims of this cross-sectional study were to determine the median UIC as a population-level indicator of iodine status among 450 rural Cambodian women farmers of reproductive age in the province of Prey Veng using single spot morning urine samples collected in 2012 and to compare population-level iodine status determined among women in Prey Veng in 2012 with the more recent national-level data from the DHS.

## 2. Materials and Methods 

### 2.1. Study Design and Population

**Figure 1:**
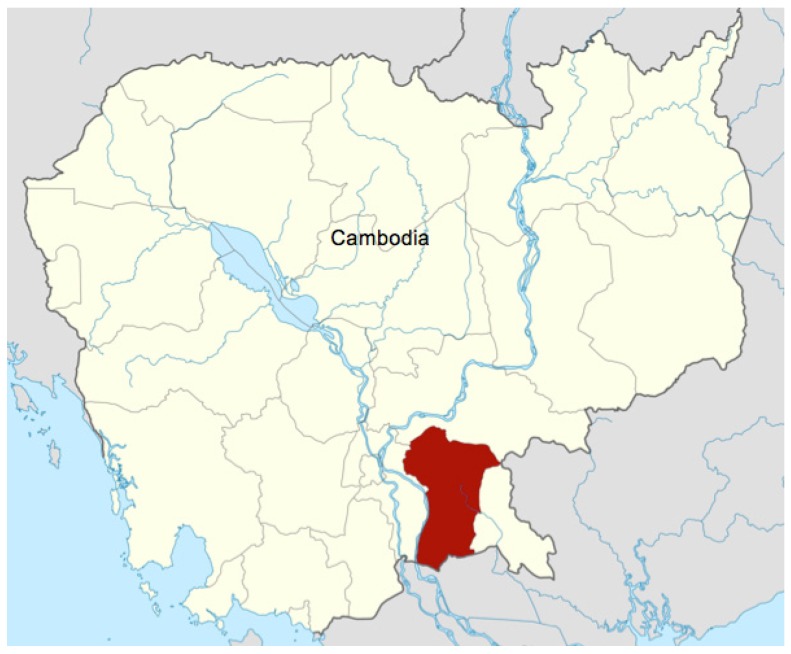
The study area of the province of Prey Veng in Cambodia.

This study used baseline data that were collected before the start of a randomized trial of homestead food production and improved aquaculture (not yet published) in 4 districts of the province of Prey Veng (Mesang, Kamchay Mear, Svay Anthor, and Bar Phnom). Prey Veng is located in Southeastern Cambodia and borders Vietnam and the east bank of the Mekong river (Figure 1). Details of recruitment and enrollment have been previously published [17]. In brief, to be eligible for the trial, women had to be between 18 and 45 years, have at least 1 child <5 years, and live in farming households with access to land and labor for agriculture and/or aquaculture activities. Ethical approval was granted (4 April 2012) by the Clinical Research Ethics Board at the University of British Columbia in Canada (H12-00451) and by the National Ethics Committee for Health Research in Cambodia. Written informed consent was obtained from women upon enrollment in the study.

### 2.2. Sample Collection, Storage, and Laboratory Analysis

A fasting single spot urine sample (~2 mL) was collected from women in the morning in June 2012. Urine samples were transported on ice from Prey Veng to the National Institute of Public Health Laboratory in Phnom Penh, Cambodia. Samples were frozen at −70 °C until shipment to Vancouver, Canada (Cryoport liquid nitrogen dry vapor shipper, Lake Forest, CA, USA) for analysis. Age, sociodemographic and other data were collected using a questionnaire administered by the Khmer speaking research staff. Current pregnancy status was self-reported.

UIC was quantified using the Sandell-Kolthoff reaction [18] with the substitution of ammonium persulfate for chloric acid as the digestive reagent as recommended by the WHO/UNICEF/ICCIDD [11] and modification for the use of 96-well plates. Seronorm™ Trace Elements Urine (SERO AS, Billingstad, Norway) was used as an analytical control for iodine concentrations.

### 2.3. Data Analysis

Mean ± SD, median, and range (min, max) UIC (μg/L) were determined for non-pregnant and pregnant women. We determined iodine status based on population median UIC [11]. We also assessed the UIC distribution frequency (*%*), which is the number of women below or above the cut-offs, or within the range, divided by the total number of women and multiplied by 100. The rationale was that comparing distribution frequencies by each category can indicate if there are large proportions of individuals with either very low or high UIC.

Statistical analysis was completed using Stata software version 13.1 for Mac (Stata Corp, College Station, TX, USA).

## 3. Results

### 3.1. Participant Characteristics

The characteristics of women in the study have been previously published [17]. In brief, a total of 93% (*n* = 420) of women were non-pregnant and 7% (*n* = 30) of women were pregnant at the time of sample collection. Mean age ± SD of women was 29.6 ± 6.5 years for non-pregnant women and 28.5 ± 4.6 years for pregnant women. Data on women’s micronutrient status (folate, iron, and vitamins B_12_ and A), inflammation status, and parity have been published elsewhere [17].

### 3.2. Urinary Iodine Concentrations and Distribution Frequencies

Table 1 presents the median UIC and distribution frequency (%) among women of reproductive age in Prey Veng, Cambodia. A urine sample was collected from 450 women at their household. One sample (*n* = 1) was missing at the time of analyses; therefore, data for only *n* = 449 are presented. Median UIC in both non-pregnant and pregnant women was indicative of adequate iodine status, for both groups (median UIC between 100–199 and 150–249 μg/L, respectively) [11]. An estimated, 28% and 37% of non-pregnant and pregnant women respectively had individual UIC <100 and <150 μg/L (cut-offs indicative of iodine deficiency [11]), suggesting that there were proportions of women in both populations that *may be* at risk of iodine deficiency as determined from the population-level assessment criteria. An estimated, 48% and 47% of non-pregnant and pregnant women, respectively, had individual UIC within the range suggestive of adequate iodine status. Excessive iodine intakes did not appear to be a problem in non-pregnant or pregnant women, as 7% and 0% of women had individual UIC ≥300 and ≥500 μg/L, respectively.

## 4. Discussion

In this cross-sectional survey in Prey Veng, Cambodia, we found the median UIC of rural non-pregnant and pregnant women using spot morning urine samples collected in 2012 were indicative of adequate iodine status for both groups (median UIC between 100–199 and 150–249 μg/L, respectively) [11]. A previous study in Cambodian children in the province of Kampong Speu using data collected in 2012, showed evidence of adequate to more than adequate iodine status in school-aged children [15]. As compared to Prey Veng, located in southeastern Cambodia bordering Vietnam, Kampong Speu is in central Cambodia about 60 km west of the capital city, Phnom Penh. Conversely, the most recent DHS in Cambodia using nationally representative data collected in 2013–2014 revealed that large proportions of women (78%) and children under 5 years (66%) had UIC <100 μg/L, suggesting mild or moderate deficiency [16].

One of the many factors that may be contributing to differences observed in the iodine status of children in these studies is geographical location. In this regard, a recent study using data collected in 2014 by Laillou *et al.* [19] revealed that more than 60% of all household salt sampled in Cambodia was not adequately iodized and that there were substantial differences in the proportion of iodized-salt across provinces. For example, in Prey Veng, ~40%–59.9% of salt was not adequately iodized, as compared to ~20%–39.9% in Kampong Speu, and less than 20% in Siem Reap. In the provinces of Battambang, Pursat and Takeo, proportions of inadequately iodized salt were as high as 60%–79.9%.

Laillou *et al.* suggested that new policy changes in the national salt iodization program, enforced in 2010, were likely a driving force for the increasing prevalence of non-iodized salt in Cambodia, as observed in his study. These policy changes included the transfer of responsibility for the procurement and supply of potassium iodate (the ingredient used to fortify salt with iodine) from UNICEF to the salt producers themselves, with the goal to stimulate ownership of and increase the long-term sustainability of the iodization programs in Cambodia [19]. Laillou *et al.* suggest that the lack of appropriate monitoring and enforcement of the iodization programs by the government could also be contributing to the limited success of the national salt iodization program. Further, the current iodization standard in Cambodia is to use 30–59.9 mg iodine per kg of salt. In many other countries, standards are lower (e.g., 15–30 mg/kg). It has been suggested that, if the standards in Cambodia were reduced to ~15–30 mg/kg, the salt producers may be more compliant to adequate iodization methods [19].

One limitation of our study is that it is unknown whether any of the 420 non-pregnant women in our study were lactating at the time of sample collection. Given that one criterion of our study was that women had at least 1 child <5 years, it is likely that many of these women were currently lactating at the time of sample collection. However, the WHO/UNICEF/ICCIDD cut-offs indicative of iodine deficiency for non-pregnant and lactating women are both 100 μg/L; therefore, whether or not a woman was lactating would not affect the interpretation in our study population. Interestingly, the recent DHS (2013–2014) reports the percent distribution of UIC for mothers 15–49 years that had given birth since January 2009 (*n* = 737); thus, similar to the current study, results are also generalizable to women with a parity of ≥1, as the study excluded women who had never given birth. Although the median UIC is not reported, the value could be inferred from data based on the reported distributions. Additionally, it is not clear if any of the mothers in the recent DHS survey were either pregnant or lactating at the time of sample collection. If pregnant women were included, population-level iodine status may be slightly overestimated (*i.e.*, interpreted as less deficiency) [9].

We also acknowledge the limitation that our sample is only representative of one province in Cambodia. We had a small sample size of pregnant women (*n* = 30); hence, we caution that our findings in pregnant women may be biased to high variation in UIC [11]. In assessment of larger populations, this variation in UIC is thought to “level-out” [1] and has less of an influence on median values. Further, we do not know the trimester of pregnancy or gestational age of the 30 pregnant women in the study. This is important because the higher UIC observed in pregnant women, as compared to non-pregnant women, may be caused by an increased renal excretion of iodine, which can occur in pregnancy (specifically in the 2nd and 3rd trimesters) as a result of hemodilution and/or an increased glomerular filtration rate [20]. The consumption and timing of prenatal supplements that include iodine are also important for consideration when using spot urine samples [21]. We did not collect data on the consumption of multiple micronutrient supplements that include iodine, but we note that it is not common practice in Cambodia for women to consume these. Rather, women consume iron and folic acid supplements, if any [22]; hence, we speculate that iodine intake from supplements was not a confounding factor among pregnant women in our study.

## 5. Conclusions

We conclude that non-pregnant and pregnant women in rural Prey Veng, Cambodia had adequate iodine status based on spot morning urine samples collected in 2012. A 2007 WHO Technical Consultation Report [9] recommends that iodine status should be assessed in surveys every 3–5 years. Accurate nation-wide data on population-level iodine status is essential to determine if universal salt iodization programs are effective and achieving adequate coverage. More research is warranted to determine population-level iodine status among a larger and more representative population of women in Cambodia, especially in light of recent policy changes to the national program for universal salt iodization and the conflicting findings from the limited number of previous surveys in women and children.

## Figures and Tables

**Table 1:** Urinary iodine concentrations (μg/L) and distribution frequencies of women (18–45 years) in Prey Veng, Cambodia for non-pregnant and pregnant women ^1^.

Non-Pregnant		Pregnant	
Number, *n (%)*	419 (93.3)	Number, *n (%)*	30 (6.7)
Median, μg/L	139 ^2^	Median, μg/L	157 ^2^
Range (min, max), μg/L	992 (0, 992)	Range (min, max), μg/L	326 (49, 375)
UIC distribution frequency ^3^		UIC distribution frequency ^3^	
<20 μg/L	1.2%	<20 μg/L	0%
<50 μg/L	6.4%	<50 μg/L	3.3%
<100 μg/L	28.4%	<150 μg/L	36.7%
100–199 μg/L	47.7% ^4^	150–249 μg/L	46.7% ^4^
200–299 μg/L	17.2%	250–499 μg/L	16.7%
≥300 μg/L	6.7%	≥500 μg/L	0%

^1^ Total *n* = 449 (*n* = 1 sample was missing at the time of analysis). UIC: urinary iodine concentration; ^2^ Median UIC are indicative of adequate iodine status for both non-pregnant women (100–199 μg/L) and pregnant women (150–249 μg/L) [11]; ^3^ distribution frequency (%) is the number of women below or above the cut-offs, or within the range, divided by the total number of women and multiplied by 100; ^4^ proportion of women with individual UIC within the range suggestive of adequate iodine status [11].

## Abbreviations

DHS: Demographic and Health Survey
FAO: Food and Agriculture Organization of the United Nations
ICIDD: International Council for Control of Iodine Deficiency Disorders
IDD: Iodine Deficiency Disorders
RNI: Recommended Nutrient Intakes
UIC: Urinary Iodine Concentration
UNICEF: United Nations Children’s Fund
USI: Universal Salt Iodization
WHO: World Health Organization
